# Supplementary material for: ProteinShader: illustrative rendering of macromolecules
Source: BMC Struct Biol. 2009 Mar 30;9:19. doi: 10.1186/1472-6807-9-19 (PMC2672931; doi:10.1186/1472-6807-9-19)
Supplement: Additional file 1 — ProteinShader program without source code. This compressed file contains the complete ProteinShader program including associated libraries, but no source code. A README.txt file gives an overview of the ProteinShader distribution, and the index.html file in the help subdirectory has directions on getting started with the program as well as a set of tutorials. [file 1472-6807-9-19-S1.zip › ProteinShader-beta-0_9_4-binary/help/api/org/proteinshader/structure/class-use/Residue.html]

Uses of Class org.proteinshader.structure.Residue (ProteinShader API)


|  |  |  |
| --- | --- | --- |
| **Overview** \| **Package** \| **Class** \| **Use** \| **Tree** \| **Deprecated** \| **Index** \| **Help** | |  |
| PREV   NEXT | **FRAMES**    **NO FRAMES**     **All Classes** |  |


---


## **Uses of Class org.proteinshader.structure.Residue**

| Packages that use Residue | |
| --- | --- |
| **org.proteinshader.gui** | Holds all of the Swing GUI components and their associated listeners, including class Renderer, which is registered as a listener for the GLCanvas object that is used a drawing surface. |
| **org.proteinshader.gui.components.controlpanel** | Holds the JPanels used in the right-side retractable control panel of the GUI. |
| **org.proteinshader.structure** | Holds the classes that store information from a Protein Data Bank file: Structure, Model, Chain, AminoAcid, Heterogen, Water, Atom, Bond, Helix, BetaStrand, Loop, *etc*. |

Uses of Residue in org.proteinshader.gui

| Methods in org.proteinshader.gui that return Residue | |
| --- | --- |
| `Residue[]` | `MediatorImpl.getCurrentResidues()`             Returns the current Residues. |
| `Residue[]` | `Mediator.getCurrentResidues()`             Returns the current Residues. |

| Methods in org.proteinshader.gui with parameters of type Residue | |
| --- | --- |
| `void` | `MediatorImpl.setCurrentResidues(Residue[] residues)`             Sets the current Residues. |
| `void` | `Mediator.setCurrentResidues(Residue[] residues)`             Sets the current Residues. |

Uses of Residue in org.proteinshader.gui.components.controlpanel

| Methods in org.proteinshader.gui.components.controlpanel that return Residue | |
| --- | --- |
| `Residue` | `SelectorPanel.getSelectedResidue()`             Returns the currently selected Residue from the Residue list. |
| `Residue[]` | `SelectorPanel.getSelectedResidues()`             Returns an array of the currently selected Residues from the Residue list. |

| Methods in org.proteinshader.gui.components.controlpanel with parameters of type Residue | |
| --- | --- |
| `int` | `SelectorPanel.addAtomsToAtomList(Residue residue)`             Adds all Atoms in the Residue to the Atom list. |
| `void` | `SelectorPanel.setAtomList(Residue residue)`             Sets the Atom list for a Residue. |

Uses of Residue in org.proteinshader.structure

| Subclasses of Residue in org.proteinshader.structure | |
| --- | --- |
| `class` | `AminoAcid`             This concrete subclass of the abstract class Residue stores information on an amino acid in a PDB structure entry. |
| `class` | `Heterogen`             This concrete subclass of the abstract class Residue stores information on a heterogen in a PDB structure entry. |
| `class` | `Water`             This concrete subclass of the abstract class Residue stores information on a water molecule in a PDB structure entry. |

| Methods in org.proteinshader.structure that return Residue | |
| --- | --- |
| `Residue` | `Chain.getResidue(String residueID)`             Returns the Residue with the residueID given as an argument. |
| `Residue` | `Structure.getResidue(String residueID, String chainID, String modelID)`             Returns the Residue with the IDs given as arguments. |

---


|  |  |  |
| --- | --- | --- |
| **Overview** \| **Package** \| **Class** \| **Use** \| **Tree** \| **Deprecated** \| **Index** \| **Help** | |  |
| PREV   NEXT | **FRAMES**    **NO FRAMES**     **All Classes** |  |


---

# *Copyright © 2007-2008*
